# Spatial and Temporal Variation in Fungal Endophyte Communities Isolated from Cultivated Cotton (*Gossypium hirsutum*)

**DOI:** 10.1371/journal.pone.0066049

**Published:** 2013-06-11

**Authors:** María J. Ek-Ramos, Wenqing Zhou, César U. Valencia, Josephine B. Antwi, Lauren L. Kalns, Gaylon D. Morgan, David L. Kerns, Gregory A. Sword

**Affiliations:** 1 Department of Entomology, Texas A & M University, College Station, Texas, United States of America; 2 Department of Soil and Crop Sciences and Texas AgriLife Extension, Texas A & M University, College Station, Texas, United States of America; 3 AgriLife Extension Service, Texas A & M University, Lubbock, Texas, United States of America; Nanjing Agricultural University, China

## Abstract

Studies of fungi in upland cotton (*Gossypium hirsutum*) cultivated in the United States have largely focused on monitoring and controlling plant pathogens. Given increasing interest in asymptomatic fungal endophytes as potential biological control agents, surveys are needed to better characterize their diversity, distribution patterns and possible applications in integrated pest management. We sampled multiple varieties of cotton in Texas, USA and tested for temporal and spatial variation in fungal endophyte diversity and community composition, as well as for differences associated with organic and conventional farming practices. Fungal isolates were identified by morphological and DNA identification methods. We found members of the genera *Alternaria*, *Colletotrichum* and *Phomopsis*, previously isolated as endophytes from other plant species. Other recovered species such as *Drechslerella dactyloides* (formerly *Arthrobotrys dactyloides*) and *Exserohilum rostratum* have not, to our knowledge, been previously reported as endophytes in cotton. We also isolated many latent pathogens, but some species such as *Alternaria tennuissima*, *Epicoccum nigrum*, *Acremonium alternatum*, *Cladosporium cladosporioides*, *Chaetomium globosum* and *Paecilomyces* sp., are known to be antagonists against plant pathogens, insects and nematode pests. We found no differences in endophyte species richness or diversity among different cotton varieties, but did detect differences over time and in different plant tissues. No consistent patterns of community similarity associated with variety, region, farming practice, time of the season or tissue type were observed regardless of the ecological community similarity measurements used. Results indicated that local fungal endophyte communities may be affected by both time of the year and plant tissue, but the specific community composition varies across sites. In addition to providing insights into fungal endophyte community structure, our survey provides candidates for further evaluation as potential management tools against a variety of pests and diseases when present as endophytes in cotton and other plants.

## Introduction

Fungal endophytes are fungi that internally colonize plant tissues without causing evident damage or disease [1]. Several groups have proposed that fungal endophytes evolved from plant pathogenic fungi that have long latent periods, or have lost their virulence [2]–[3]. In addition, there are examples of specific environmental conditions triggering pathogenicity of previously asymptomatic endophytes [1] [4]. Alternatively, a number of studies suggest that fungal endophytes can be involved in many beneficial interactions with their hosts, providing protection against a variety of stressors including herbivores, pathogens, heat and drought [1] [5]–[13]. Studies of beneficial fungal endophytes present in agricultural crops have focused on the analysis of their assemblages *in planta*, physiological interactions with host plants, the production of secondary fungal metabolites, and their potential use in biological control of plant diseases and insects [1] [8] [14]–[19]. Some studies have shown positive effects of these fungal endophytes on plant growth including higher rates of germination and rooting, and increased tissue biomass and seed production under adverse conditions [20]–[24].

Fungal endophyte surveys explicitly aimed at isolating candidate beneficial fungal endophytes from cotton (*Gossypium hirsutum*) cultivated in the United States have not been reported to date. However, many studies dating back to the 1920s have been published on the identification of fungi isolated from a variety of cotton tissues, primarily with an emphasis on monitoring fungal diseases [25]–[29]. Among the species identified in these studies, several have since been found to live as endophytes in healthy plants across a range of different species [30]. It seems likely that at least some of the fungi previously isolated and considered as pathogenic (or at least putatively pathogenic) in cotton may be more appropriately considered as asymptomatic endophytes. Thus, fungal endophyte community studies have the potential to provide an unexplored source of candidate strains for potential beneficial applications.

Recent surveys of fungal endophytes in healthy cotton conducted in Australia and Brazil demonstrated the presence of a diversity of species and broad scale variation in community composition. McGee (2002) [31] surveyed healthy leaves obtained from a cotton-breeding trial in Australia and identified fungal endophytes from ten different genera by morphological identification of their fruiting bodies. Among the genera obtained, an isolate of *Phomopsis* sp. showed promising effects in reducing caterpillar herbivory. Although *Phomopsis* sp. has been reported as a plant pathogen [32], only rarely does it cause disease [26] [31] [33]. Another Australian study by Wang et al. (2007) [34] sought to determine whether fungal endophytes of native *Gossypium* species could be pathogenic to cultivated cotton, *Gossypium hirsutum*. They showed that fungal endophytes were common in four native *Gossypium* species and dominated by six genera including *Phoma*, *Alternaria*, and *Fusarium.* Interestingly, none of these isolates caused disease symptoms when inoculated to cotton under controlled conditions [34]. A more recent study conducted in Brazil compared fungal endophyte communities associated with transgenic and non-transgenic cotton [35]. Their results indicated that the endophytic fungal community was not affected by the expression of Cry1Ac protein from *Bacillus thuringiensis* (*Bt*) in transgenic cotton plants [35]. In total, they isolated 17 genera of endophytes from both *Bt* and non-*Bt* cotton including *Xylaria* sp., *Phoma* sp., *Phomopsis* sp., *Lecanicillium* sp., *Tritirachium* sp., *Pestatiopsis* sp., *Cladosporium* sp., *Fusarium* sp. and *Guignardia* sp. among others [35].

In order to better characterize communities and functional roles of fungal endophytes in cotton and isolate strains with potential beneficial applications, we conducted a survey of endophytes in asymptomatic cotton in Texas, USA. We focused on commercial varieties cultivated at eight sites distributed across two ecologically-distinct growing regions in north and central Texas, each sampled at two different times of the season. We also surveyed cotton grown on organic farms in comparison to conventional farms. We found a total of 69 different endophytic fungal taxa grouped into 44 different genera. We found evidence of differences in community composition depending on temporal variation and plant tissue rather than on location or cultivation practices. Among the fungal endophytes isolated, several are candidates for potential use as beneficial endophytes in the management of plant pathogens, insect pests and nematodes based either on their known effects as endophytes in other plants or their ecological roles outside the plant.

## Results

### Endophyte Isolation and Identification

**Figure 1:**
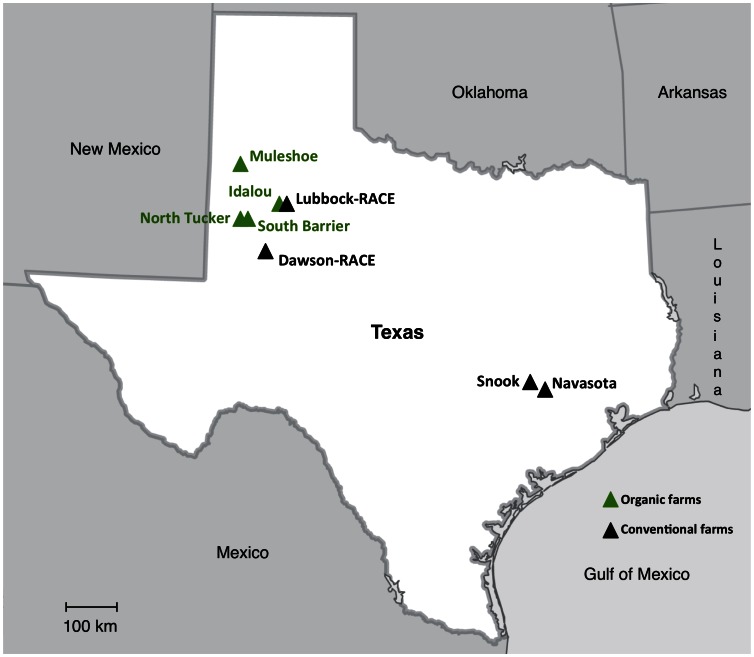
Map of survey area. Geographic location of the eight different cotton farms surveyed during the 2011 growing season in Texas, USA. Green symbols correspond to farms managed following organic practices and black symbols correspond to farms managed using conventional practices.

**Figure 2:**
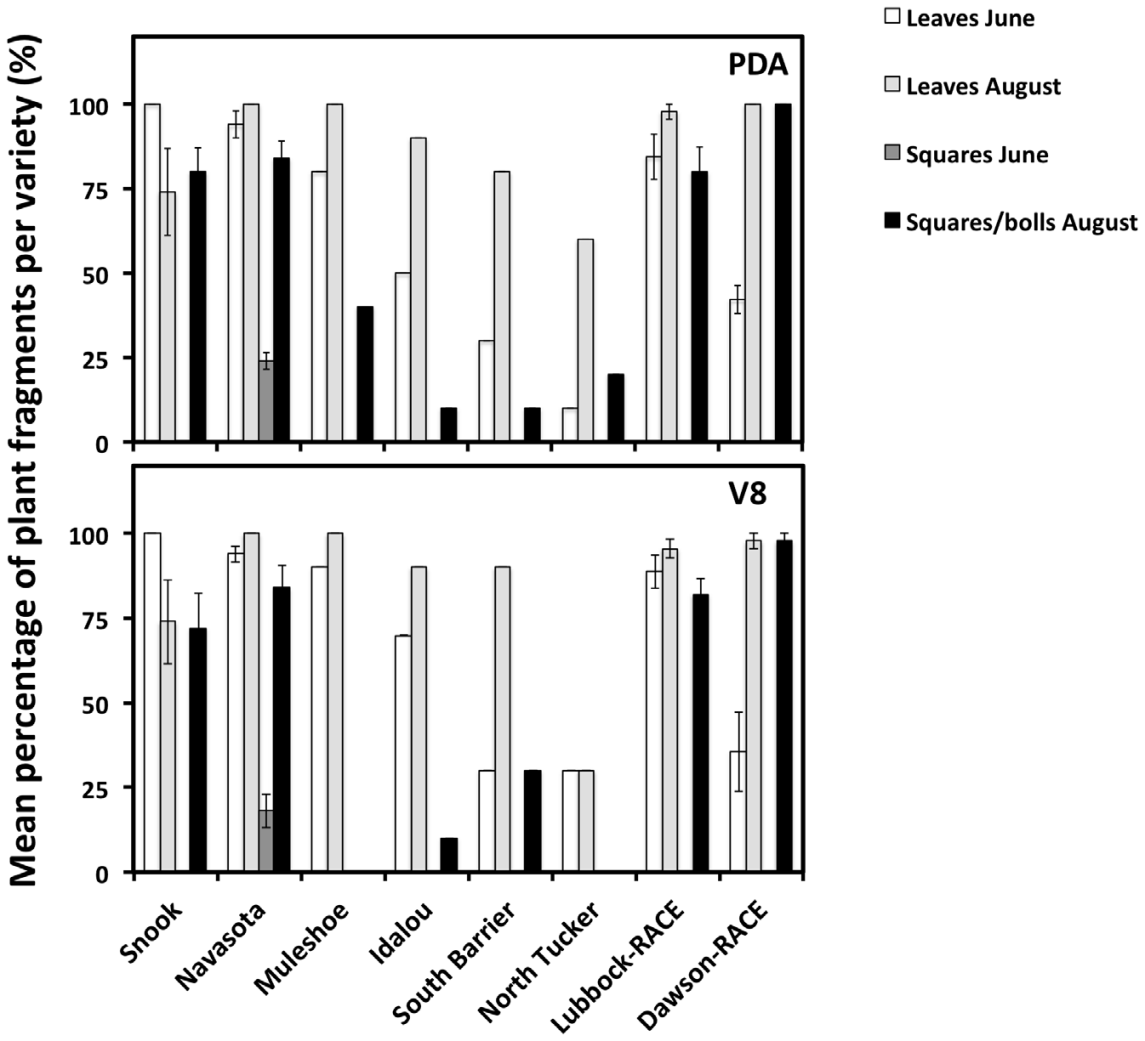
Fungal endophyte isolation efficiency. Fungal endophyte taxa isolation efficiency expressed as the mean % of plant fragments (leaves, squares and bolls as explained in materials and methods) per variety at each site from which at least one endophytic isolate was obtained. Sets of data are also grouped by time of the season and tissue surveyed. (A) Isolates obtained using PDA media. (B) Isolates obtained using V8 media.

The surface sterilization protocol was stringent enough to eliminate epiphytic fungi, as neither fungi nor bacteria grew after making tissue imprints onto the surface of PDA and V8 media control plates. The total number of endophytic isolates obtained from leaves collected in June 2011 was 1259, and from squares that were present at the time at only one locality, Navasota, it was 46. Later in the season in August, the total number of isolates obtained from leaves was 1354, and from squares/bolls it was 802. Endophytic fungi were isolated from plant samples at different frequencies depending on the tissue, variety, location and cultivation practices surveyed (Figures 1 and 2; Table 1). One way ANOVA (SPSS 20.0, IBM North America, New York, USA) of isolation percentages normalized using arcsine transformation [36] indicated that the number of isolates recovered varied significantly by tissue (F_249, 248_ = 8.321, *P*<0.05), time of the season (F_249, 248_ = 5.142, *P*<0.05), location (F_249, 242_ = 17.925, *P*<0.05), cultivation practices (F_249, 248_ = 80.384, *P*<0.05) and cotton variety (F_249, 233_ = 5.414, *P*<0.05), but was independent of culture media used (F_249, 248_ = 0.054, *P* = 0.816). The highest isolation percentages of approximately 100% were obtained from leaves collected in August 2011 in fields under conventional cultivation practices such as Snook, Navasota, Lubbock-RACE and Dawson-RACE. The lowest isolation frequencies (<30%) were obtained from squares/bolls from all organic farms (Muleshoe, Idalou, South Barrier and North Tucker) (Figure 2). Fungal isolates obtained were identified using ITS1 sequence and morphological data (Table 2) resulting in a total of 69 different endophytic fungal taxa (OTUs) grouped into 44 different genera.

**Table 1:** Location and cotton variety information for the farms surveyed during the 2011 season.

Location	Cultivation	GPS coordinates	Elevation (m)	Varieties sampled	Sampling dates
Snook, TX	Conventional	N30°31.588’,W96° 28.078’	74.98 m	NG4012B2RF ST4498B2F PHY499WRF FM1740B2F DP1044B2F	June 15^th^ and July 24^th^
Navasota, TX	Conventional	N30°23.835’,W96° 14.376’	61.26 m	PHY375WRF FM1740B2F DP1044B2RF DP0912B2RF 11R115B2R2	June 16^th^ and July 25^th^
Muleshoe, TX	Organic	N34°16.380’,W102° 22.958’	102.41 m	FM958	June 21^st^ and August 1^st^
Idalou, TX	Organic	N33°40.698’,W101° 38.115’	106.68 m	FM975	June 21^st^ and August 1^st^
South Barrier, TX	Organic	N33°18.035’,W102° 25.050’	101.19 m	FM958	June 21^st^ and August 1^st^
North Tucker, TX	Organic	N33°17.750’,W102° 22.820’	103.32 m	FM958	June 21^st^ and August 1^st^
Lubbock-RACE, TX	Conventional	N33°35.707’,W101° 33.877’	103.02 m	AT81220B2RF NG4010B2RF FM2484B2F PHY499WRF CG3787B2RF	June 22^nd^ and August 2^nd^
Dawson-RACE, TX	Conventional	N32°46.516’, W101°56.547’	102.41 m	AT81220B2RF NG4012B2RF FM2484B2F PHY367WRF CG3787B2RF	June 22^nd^ and August 2^nd^

**Table 2:** Identification of fungal endophyte taxa isolated from cotton cultivated in Texas in June-August 2011.

Sequence accession Genbank number	Fungal taxa	Isolates	Sequence accession Genbank number	Fungal taxa	Isolates
KC800871	*Acremonium alternatum*	1	KC800875	*Colletotrichum capsici*	1
KC800839	*Alternaria alternata*	1	KC800892	*Coniolariella gamsii*	1
KC800833	*Alternaria brassicae*	6	KC800870	*Coniothyrium aleuritis*	1
KC800836	*Alternaria compacta*	2	KC800862	*Coniothyrium* sp.	2
KC800844	*Alternaria dianthi*	1	KC800885	*Corynespora cassiicola*	1
KC800829	*Alternaria longipes*	5	KC800865	*Diaporthe* sp.	1
KC800837	*Alternaria mali*	1	KC800859	*Diatrype* sp.	1
KC800896	*Alternaria sesami*	1	KC800834	*Drechslerella dactyloides*	1
KC800888	*Alternaria solani*	2	KC800863	*Embellisia indefessa*	1
KC800895	*Alternaria* sp.	3140	KC800886	*Epicoccum nigrum*	23
KC800894	*Alternaria tenuissima*	78	KC800831	*Epicoccum* sp.	2
KC800842	*Ascomycota* sp.	4	KC800830	*Exserohilum rostratum*	2
KC800889	*Bipolaris spicifera*	4	KC800873	*Fusarium chlamydosporum*	3
KC800881	*Cercospora canescens*	1	KC800890	*Fusarium* sp.	1
KC800866	*Cercospora capsici*	3	KC800880	*Gibellulopsis nigrescens*	4
KC800878	*Cercospora kikuchii*	1	KC800855	*Gnomoniopsis* sp.	1
KC800877	*Cercospora zinnia*	1	KC800848	*Lewia infectoria*	16
KC800876	*Chaetomium globosum*	9	KC800869	*Mycosphaerella coffeicola*	5
KC800846	*Chaetomium piluliferum*	1	KC800840	*Mycosphaerellaceae* sp.	1
KC800851	*Chaetomium* sp.	8	KC800864	*Nigrospora oryzae*	2
KC800874	*Cladosporium cladosporioides*	7	KC800891	*Nigrospora* sp.	2
KC800849	*Cladosporium* sp.	9	KC800893	*Nigrospora sphaerica*	1
KC800872	*Cladosporium uredinicola*	5	KC800867	*Paecilomyces* sp.	1
KC800838	*Cochliobolus* sp	5	KC800883	*Penicillium citrinum*	1
KC800850	*Phanerochaete crassa*	1	KC800861	*Retroconis* sp.	2
KC800857	*Phoma americana*	1	KC800832	*Rhizopycnis* sp.	1
KC800879	*Phoma subherbarum*	1	KC800860	*Schizothecium inaequale*	9
KC800882	*Phomopsis liquidambari*	1	KC800841	*Stagonospora* sp.	6
KC800853	*Phomopsis* sp.	2	KC800884	*Stemphylium lancipes*	3
KC800856	*Pleospora* sp.	3	KC800843	*Thielavia hyrcaniae*	2
KC800835	*Pleosporaceae* sp.	5	KC800845	*Thielavia* sp.	5
KC800858	*Polyporales* sp.	1	KC800852	*Ulocladium chartarum*	2
KC800854	*Preussia africana*	2	KC800868-	*Verticillium* sp.	4
KC800887	*Preussia* sp.	4	–	Unknown	24
KC816535	*Pseudozyma* sp.	1	–	Uncultured	9
KC800847	*Pyrenophora teres*	1			

### Species Richness and Biodiversity of Fungal Endophyte Communities (α-diversity)

**Figure 3:**
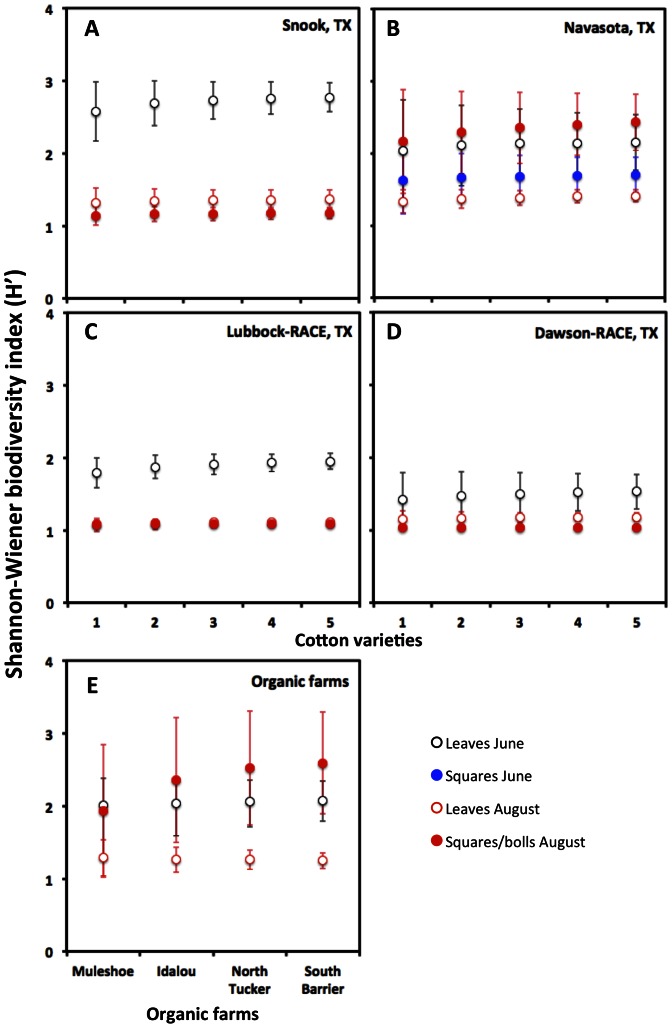
Fungal endophyte biodiversity analysis. The effects of variety and cultivation practices on fungal endophyte Shannon-Wiener biodiversity index (H’). Multiple varieties were sampled at four different sampling locations using conventional farming practices: (A) Snook, TX, (B) Navasota, TX, (C) Lubbock-RACE and (D) Dawson-RACE. Only a single variety was grown at each of the four sampled organic farms (E). Refer to Table 1 for the specific varieties sampled at each site.

**Figure 4:**
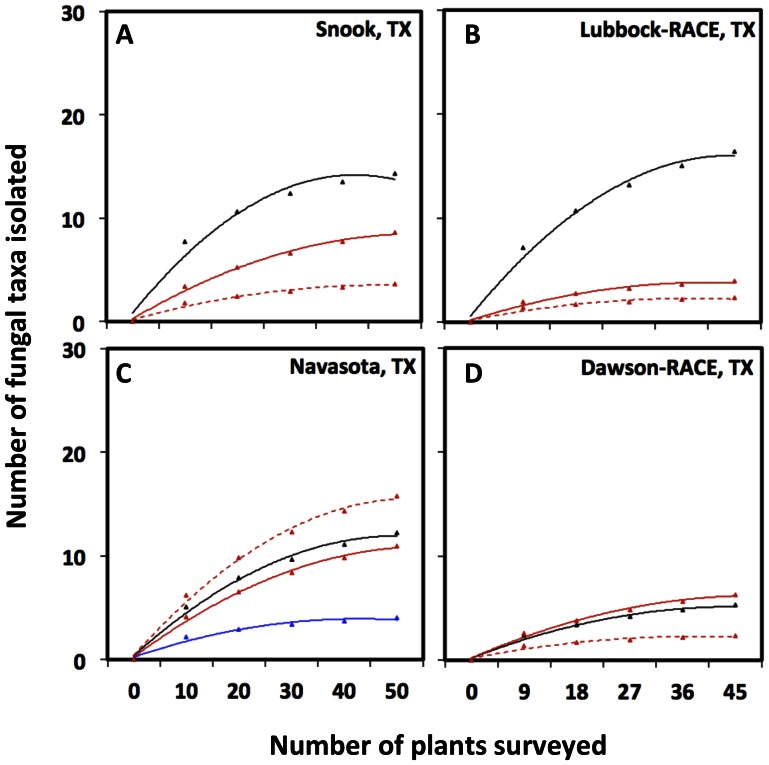
Accumulation curves of fungal taxa isolated from leaves, squares and squares/bolls. The graphs show the relationship between the number of fungal taxa isolated and the number of plants surveyed in (A) Snook, TX, (B) Lubbock-RACE, (C) Navasota, TX and (D) Dawson-RACE. Black continuous line = Leaves surveyed in June 2011, Blue continuous line = Squares surveyed in June 2011 (Navasota, TX), Red continuous line = Leaves surveyed in August 2011 and Red dashed lines = Squares/Bolls surveyed in August 2011. Whole endophyte communities were considered per tissue and location, as cotton variety did not appear to have effect on Shannon-Wiener biodiversity indexes in the farms managed under conventional practices as shown in Figure 3.

Shannon-Wiener biodiversity values (H′) [37]–[38] compared across varieties within the four conventional farming sites where multiple varieties were sampled were not significantly different for communities isolated from a given tissue type, indicating that the number and relative abundance of recovered endophytic taxa did not vary among cotton varieties (Figure 3A–D). However, within each variety there were clear differences in endophyte diversity between tissue types and times of the season in which they were sampled (Figure 3A–D). With regards to sampling intensity, the taxa accumulation curves indicated that our sampling of 45–50 samples per site was sufficient to isolate rare fungal endophyte taxa regardless of the tissue being sampled (Figure 4). A comparison among varieties within sites practicing organic farming was not possible because only a single variety was grown at each site, but a similar pattern of variation in diversity between samples from different tissues and time of the season was observed (Figure 3E).

### Variation in Fungal Endophyte Communities Among Varieties, Tissues and Time of the Season (β-diversity)

**Figure 5:**
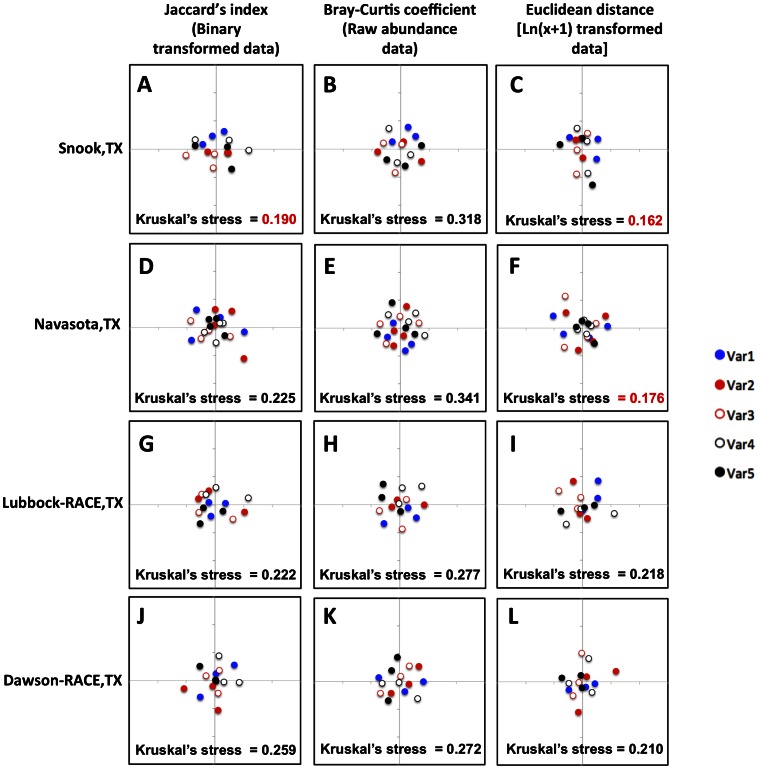
Effect of cotton variety (genotype) on fungal endophyte communities. Non-metric multidimensional scale (NMDS) plots corresponding to the clustering of endophyte communities isolated from five different commercial varieties per location indicated by different colors (see Table 1 for variety information). Each point represents a single endophyte community from a particular tissue and time of the season. For clustering analysis, three different community similarity measures were calculated: (A,D,G,J) Jaccard’s index comparing fungal taxa presence or absence among samples from each variety within a site (Binary transformed data); (B,E,H,K) Bray-Curtis coefficient which compares fungal taxa presence or absence as well as abundance among samples from each variety within a site (Raw abundance data); and (C,F,I,L) Euclidean distance using total number of fungal taxa isolated per variety [Ln (x+1) transformed data]. Kruskal’s stress values <0.2 indicating more confidence in the observed groupings are indicated in red.

**Figure 6:**
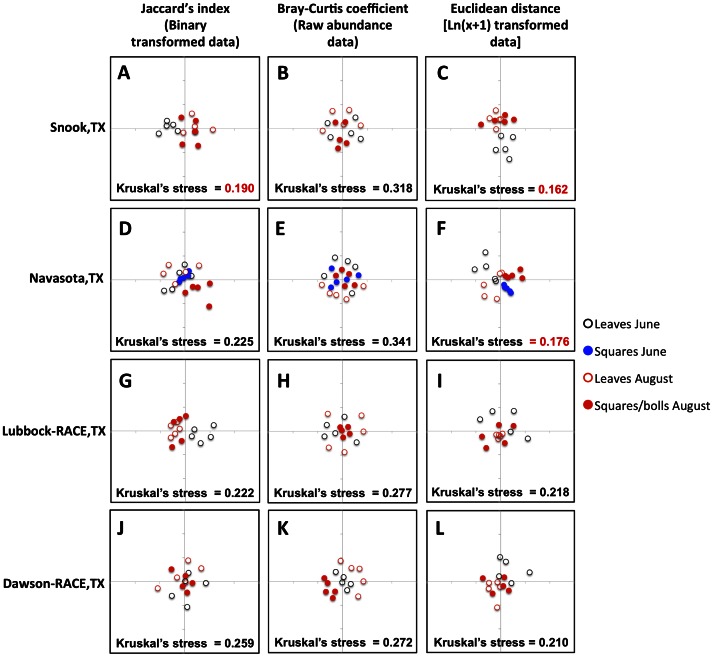
Effect of plant tissue and time of the season on fungal endophyte communities. Non-metric multidimensional scale (NMDS) plots as described for Figure 5, but labeled with different colors by tissue (Leaves, Squares and Squares/bolls) and time of the season surveyed (June and August).

Cluster analyses of endophyte community similarity measures [39] presented in two dimensional non-metric multidimensional scale (NMDS) plots [40] revealed that fungal endophyte communities did not vary among different cotton varieties at any of the sampled sites (Figure 5). However, some local endophyte communities appeared to be specific to particular tissues and times of the season (Figure 6A,C,F). Although clusters indicating similar endophyte communities were apparent depending on the tissue type and time of the season sampled at some locations, the effect was not consistent across sites (Figure 6A–L). The use of different ecological similarity measures [41] did not consistently affect the observed patterns of cluster formation (Figure 6A–L). For example, at the Snook site, clusters of similar communities according to tissue type and time of the year were observed using both the Jaccard’s index and Euclidean distance measures (Figure 6A,C), whereas at Navasota similar clustering was only observed using the Euclidean distance (Figure 6F). Kruskal’s stress values were too high (>0.2) to confidently discern any clustering of communities at the other sites sampled regardless of the distance measure used (Figure 6G–L).

We could not analyze the effect of variety at the four organic farms we surveyed (Muleshoe, Idalou, South Barrier and North Tucker) because there was only one variety cultivated at each site. As a result, the intensity of sampling and corresponding number of endophytes isolated was lower at organic sites compared to the conventional cultivated farms where five varieties were sampled, precluding direct comparisons. Therefore, we focused on only identifying the fungal endophyte taxa isolated at the organic farms. Results indicated that there were no unique species of fungal endophytes isolated only from organic farms.

**Figure 7:**
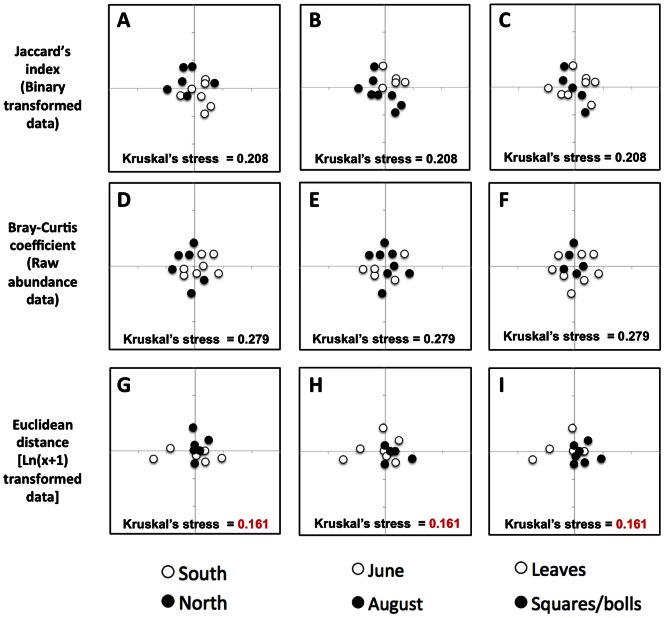
Effect of region, time of season, and tissue on whole fungal endophyte communities. Non-metric multidimensional scale (NMDS) plots corresponding to the clustering of endophyte communities grouped by location (North: Lubbock-RACE and Dawson-RACE; South: Snook and Navasota), time of the season (June and August) and tissues surveyed (Leaves, Squares and Squares/bolls). Analysis was done as explained in Figure 5, but whole endophyte communities per site were obtained by grouping taxa from all five varieties sampled at each location.

Given that we did not find any effect of cotton variety on fungal endophyte community composition within sites (Figures 3 and 5), we grouped all fungal endophyte taxa obtained at each site and then re-calculated Jaccard’s indexes, Bray-Curtis coefficients and Euclidean distances to examine regional variation across sites (Figure 7). We did not observe obvious regional clustering of fungal endophyte communities isolated from the farms located in the Southern Blacklands region of Texas (Snook and Navasota) relative to the communities isolated from farms located to the north in the Southern High Plains region of Texas (Lubbock-RACE and Dawson-RACE) (Figure 7A,D,G). Nor did we observed clustering of fungal endophyte taxa communities isolated from leaves compared to those from squares/bolls (Figure 7C,F,I). The only suggestive pattern of community similarity when considering variation across all sites was in the endophyte communities sampled at different times of the season and compared using Jaccard’s index (Figure 6B). However, confidence in this pattern was marginal (Kruskal’s stress = 0.208). Increasing the dimensionality of the NMDS analyses increased the confidence in the observed clustering patterns in all the cases, but did not change the interpretation of community similarity patterns as already shown in the two dimension plots (Figure S1).

## Discussion

Plants in both natural and agricultural settings can host diverse endophyte communities. All plants surveyed to date contain fungal endophytes, and many isolates have been obtained from a broad range of plants including trees [42]–[43], palms [44]–[45], grasses [46], sea grasses [47], crops [30] and lichens [48]. Endophytes have been referred to as a hidden component of fungal diversity [12] [49]–[50]. The diversity of endophytic fungi within a given plant can be substantial with reports of up to 22 species in a single leaf of the tropical tree *Manilkara bidentata* [1] [51]–[52] and 51 different operational taxonomic units (OTUs) associated with roots of the arid grassland grass *Bouteloua gracilis* [53]. The aim of this study was to characterize the fungal endophytes present in cotton and to analyze their spatial and temporal patterns of variation (Figure 1; Table 1). There have been many systematic studies investigating *in planta* microbial communities in cotton in the United States dating back to the 1920’s (*e.g.*, [25]). However, most have focused on identifying plant pathogens, and studies examining the factors underlying variation in fungal endophyte communities have been relatively rare (*e.g.*, [29] [35]).

The study of endophytes is a methods-dependent process [54]–[55] with the identity and range of isolates obtained potentially influenced by a number of experimental variables that, in turn, can affect the comparability of endophyte datasets. Highly stringent surface sterilization protocols can potentially kill fungal endophytes [56], thereby reducing the ability to detect viable isolates grown on media. Our surface sterilization method was stringent enough to eliminate viable fungal pathogens and epiphytes living on the surface of surveyed tissues [57]–[58], as indicated by the lack of microbial growth on control PDA and V8 media following tissue imprints of sterilized plant fragments. However, the high number of endophytic fungal isolates cultured from many samples (Figure 2) suggests that the surface sterilization procedure did not systematically kill endophytic fungi. Our use of PDA and V8 media may also have affected the number of isolates obtained if they were unsuitable for certain taxa. Given that the same sterilization procedure, growth media and incubation conditions were applied to all samples collected for this study, our comparisons of communities across varieties, times of the season and tissue type should be unaffected by any bias related to the specific fungal isolation methods.

The 69 fungal endophyte OTUs isolated in this survey (Table 2), largely corresponds to the numbers obtained in similar studies of cotton and many other crops [29]–[31] [35] [52] indicating relative consistency in endophyte isolation efficiency across studies. The percentage of plant fragments yielding fungal endophytes was high in the leaves and squares/bolls surveyed in conventional farms, and in the leaves surveyed in two of the organic cultivated farms (Muleshoe and Idalou) (Figure 2). We did not detect significant differences in isolation percentage depending on culture media used. However, there were significant differences in isolation percentage depending on time of the year and tissue surveyed. In the organic farms, fewer plant fragments yielded endophytic isolates overall relative to those from conventional farms. This suggests that specific organic farm practices may influence the prevalence of fungal endophytes. However, since different cotton varieties were grown in the organic and conventional farms, an effect of plant genotype cannot be ruled out. Future studies of endophytes from the same cotton variety grown under both organic and conventional cultivation practices will be required to address this possibility.

It is of interest to note that an overwhelming majority (93.5%) of the isolates recovered in this survey were members of the genus *Alternaria*. Isolates of some species such as *A. alternata* and *A. macrospora* are cotton pathogens [59]–[60]. We recovered only one positively identified isolate of *A. alternata* and no *A. macrospora*. However, we were not able to identify most of the *Alternaria* isolates to the species level and cannot rule out that they may be pathogens. Previous studies have shown symptomless *Alternaria* infections in cotton [60] and variation among cultivars in susceptibility exists [59]. Thus, the abundance of *Alternaria* sp. that we recovered may reflect either the presence of many non-virulent species or strains, or latent pathogens and the absence of the environmental conditions that induce symptoms [60].

The diversity of endophytic fungi in a single host species can vary both temporally and geographically. In addition, fungal taxa composition can also depend on multiple factors such as plant density, nutrient availability, local environmental conditions and interaction with soil fungi and bacteria [61]–[65]. The Shannon-Wiener biodiversity index (H′) values, which take into account the number of species (richness) and relative abundance (evenness) of the individuals present in any given sample [37]–[38], were not significantly different among varieties (Figure 3). However, we did observe differences in H′ biodiversity values when they were compared within locations over time. At Snook, Lubbock-RACE and Dawson-RACE, the H′ values of fungal endophytes isolated earlier in the growing season in June were significantly higher than those of endophyte communities isolated later in August (Figure 3). However, this temporal effect was not consistent across all locations. In Navasota, Idalou, South Barrier and North Tucker, the biodiversity indexes of fungal endophytes isolated from leaves surveyed in August were significantly lower that the leaves surveyed earlier in June and the squares/bolls isolated in August (Figure 3). Interestingly, in Navasota, the H′ values were not different between squares and leaves surveyed in June. The H’ biodiversity indexes of fungal endophytes isolated in this study exhibited spatial and temporal differences consistent with previous reports [12], but there were no consistent spatial or temporal patterns of variation in α-diversity.

Within a given location, we sought to test for an effect of variety, tissue or time of the season on fungal endophyte community composition by comparing three different measures of community similarity (β-diversity), each of which incorporates different information. Jaccard’s index uses only binary presence-absence data while the Bray-Curtis coefficient incorporates quantitative species abundance data. Both measures exclude joint absences. Euclidean distance incorporates both quantitative abundance data and joint absences [39] [40]. Regardless of the community similarity measure employed, we did not observe any obvious clustering of endophyte communities associated with different cotton varieties at any of the sampled localities (Figure 5). We did observe some clustering at two sites indicating endophyte community similarity due to tissue type and time of the season, but this effect was notably inconsistent across locations (Figure 6).

We also sought to test for regional variation in fungal endophyte community composition across all locations including both conventional and organic farming practices, but could not make these comparisons within varieties because the same varieties were not grown at all sites. Given that neither the *H’* biodiversity values (Figure 3) nor the community similarity analyses (Figure 5) were affected by cotton variety, we pooled OTUs across varieties within each site to form a single endophyte community for subsequent comparisons across locations. The fungal taxa accumulation curves obtained to evaluate sampling intensity (Figure 4) indicated that our sampling of cotton at the variety trial sites [a total of 45–50 plants per location (9–10 individuals from 5 varieties)] was sufficient to isolate rare fungal endophyte taxa regardless of the tissue being assessed, but that we likely under sampled the fungal endophyte communities at the organic farm sites were only 10 plants of a single variety were sampled (Figure 4). Thus, we did not include the organic farms in our regional analysis. With respect to the effect of organic farming practices on cotton fungal endophyte communities, it will be necessary to increase the intensity of sampling and compare different cotton varieties cultivated in those sites to know more about the ecology and distribution of any fungal endophytes that might be specifically affected by organic farming practices.

Evaluating whole fungal endophyte community composition across all conventional farming sites where multiple varieties were grown did not reveal any strong pattern of similarity due to either region, time of the season or tissue type regardless of the community similarity measure used (Figure 7). The only slight clustering effect of endophyte communities observed among these farms was due to time of the season when compared using Jaccard’s similarity index (Figure 7B), indicating that the presence/absence of particular taxa played a more important role in differentiating the communities than either their relative abundances or the total number of taxa, as evidenced by the lack of clustering in the Bray-Curtis coefficient and Euclidean distance analyses (Figure 7E,H). However, confidence in the observed pattern was marginal (Kruskal’s stress = 0.208, Figure 7B), thereby moderating this conclusion. Importantly, within each of the sites surveyed, varying “site-specific” management practices were followed with the common goal of obtaining high fiber yield, (*e.g.*, irrigation, insecticide, fungicide, herbicide and fertilizer treatments). Site-specific variation in fungal endophyte communities mediated by these factors was not addressed here and will require specific manipulative experiments. We cannot rule out the possibility that variation in site-specific treatment effects may have obscured our ability to detect broad patterns in endophyte community composition.

Among the fungal endophytes that we isolated, several are candidates for evaluation for use as beneficial endophytes in cotton based on their known effects, either as endophytes or in interactions outside of the plant, against a range of insect pests, nematodes and plant pathogens (Table 2). For example, an isolate of *Acremonium alternatum* is known to have a negative effect on the moth *Plutella xylostella*, when present as an endophyte in bean [66]–[67] and to induce resistance against *Leveillula taurica*, a causal agent of powdery mildew in tomato [68]. *Chaetomium* sp. isolates are known to produce compounds displaying a wide range of antimicrobial and antitumor activities [69] and along with *Acremonium* sp. and *Paecilomyces* sp., has been shown to have a negative effect on nematodes when present as an endophyte in cucumber [70]. *Cladosporium cladosporioides* is an entomopathogenic fungus of the two spotted spider mite *Tetranychus urticae* Koch [71], but effects on arthropods as an endophyte have not been explored to date to our knowledge. Similarly, *Drechslerella dactyloides* (*Arthrobotrys dactyloides*) is known as nematode-trapping fungus in the soil and has been used as inundative biocontrol agent for nematode control in mushroom culture and against root-knot nematode on tomato [72]–[73]. However, its functional significance as an endophyte remains unexplored.

Our study highlights the potential for surveys of fungal endophtyes in cotton and other plants to reveal a rich diversity of taxa. The ecological significance and potential for use in biological control of many of them is largely unknown. Further research is required to identify the functional and ecological significance of specific endophytic fungi within the plant under different conditions or at different sites. Should any of these isolates be further developed for beneficial applications, our analysis of spatial and temporal variation in community composition provides *a priori* reason to suspect that their presence in the plant will not be restricted to specific varieties of cotton or limited to any particular locations tested here, given that we observed no effects of variety or region on fungal endophyte biodiversity or community composition. Our results indicate that the presence of fungal endophytes in cotton can be affected by both time of the year and tissue, but in the absence of any consistent patterns across sites, the factors mediating specific interactions between the fungi and plant will need to be evaluated locally.

## Materials and Methods

### Ethics Statement

Texas A&M AgriLife Extension Service officers, located in College Station, TX and Lubbock, TX provided permission/access to all eight cotton variety trial farms.

This work did not involve endangered or protected species.

### Plant Sampling

Plant tissues [asymptomatic leaves, squares (developing flowers) and bolls (fruits)] were sampled at two different times of the growing season (June and late-July/early-August) in 2011 from multiple commercial cotton varieties grown during variety trials at eight sites distributed across two distinct growing regions in north and central Texas (Figure 1; Table 1). Texas in general suffered a severe drought in 2011 with a total annual precipitation of 385.57 mm, which was critically below the normal annual precipitation of 709.17 mm (Source: National Climatic Data Center/National Oceanic and Atmospheric Administration website: http://www.ncdc.noaa.gov/temp-and-precip/ranks.php?periods%5B%5D=12&parameter=pcp&state=41&div=0&year=2011&month=12#ranks-form Accessed 2013 May 10). Although cotton is widely grown without irrigation in Texas, only cotton grown at irrigated sites was sampled for this study.

Plants (N = 9–10 individuals per variety) were randomly sampled across multiple replicate plots (in variety trials) to account for within-field spatial variation. Asymptotic tissues from apparently healthy plants were collected to reduce the chance of sampling pathogenic fungi (one leaf, and when present, one square and one boll per plant). For leaf samples, the 5^th^ true leaves of young plants early in the season and leaves at the top of the canopy of mature plants later in the season were collected. The first developing flower buds (referred to as squares) were collected from young early season plants when present, and the squares at the top were collected from mature plants. Immature fruits (bolls) were collected from the middle of mature plants later in the season. Samples were stored in individual sealed plastic bags and kept refrigerated until processed in the lab.

### Endophyte Isolation

Using a laminar flow cabinet as a sterile workspace, plant samples were rinsed in tap water and surface sterilized by immersion in 70% ethanol for 5 min, 10% bleach solution for 3 min, and rinsed twice with autoclaved distilled water. Samples were blotted dry using autoclaved paper towels. Five individual surface sterilized leaves, squares and bolls (N = 15 total samples) were randomly selected and imprinted onto fresh potato dextrose agar (PDA) and V8 media as a way to monitor surface sterilization efficiency. For endophyte isolation, leaves were cut in small fragments of approximately 1 cm^2^. Squares and bolls were cut in six pieces. Any fiber present was removed and cut into six smaller pieces. Leaf fragments were placed upside down on PDA and V8 medium plates in triplicate. Each plate contained 3 leaf fragments for a total of 9 fragments assayed per plant. For squares collected early in the season, 3 slices per square were plated on PDA and V8 media as with the leaf fragments. Because of similarity in size and location within a plant, when collected later in the season, squares and bolls from a given plant were plated together on petri dishes containing two square slices, two boll slices and two pieces of fiber. Antibiotics Penicillin G (100 Units/mL) and Streptomycin (100 µg/mL) (Sigma, St Louis, MO, USA) were added to the media to suppress bacterial growth. All plates were incubated in the dark at room temperature for, in average, two weeks until growth of fungal endophyte hyphae from plant tissues was detected.

### Endophyte Identification

We used an inclusive combination of morphological and molecular fungal endophyte identification. Once fungal hyphae were detected growing from the plant material, samples were taken to obtain pure fungal isolates. For identification by PCR, genomic DNA was extracted from mycelium of each isolated fungal strain, following a chloroform:isoamyl alcohol 24∶1 protocol [74] and fungal specific primers were used to amplify the ITS (Internal Transcribed Spacer) region of nuclear ribosomal DNA [75]–[78]. This region is the primary barcoding marker for fungi [77] and includes the ITS1 and ITS2 regions, separated by the 5.8S ribosomal gene. In order to avoid introducing biases during PCR (taxonomy bias and introduction of mismatches), it has been suggested to amplify the ITS1 region only [77], therefore the primers ITS1 (5′ TCC GTA GGT GAA CCT GCG G 3′) and ITS2 (5′ GCT GCG TTC TTC ATC GAT GC 3′) were used to amplify and sequence the ∼240 bp ITS1 region of each one of our isolated fungal strains. Sequencing was conducted at the Texas A & M University College of Veterinary Medicine & Biomedical Sciences sequencing facility and Macrogen Corp., Maryland, USA. The resulting sequences were aligned as query sequences with the publicly available databases GenBank nucleotide, UNITE [79] and PlutoF [80]. The last two are specifically compiled and used for fungi identification. In all the cases, the strains were identified to species level if their sequences were more than 95% similar to any identified accession from all three databases analyzed [81]. When the similarity percentage was between 90–95%, the strain was classified at genera, family, order, class, subdivision or phylum level depending on the information displayed in databases used. In addition, some of the isolates had lower similarity values (from 30–90%) and were classified as unknown or uncultured depending on the information displayed after BLAST analysis. In total, sequences from 69 unique fungal endophyte taxa were identified (Table 2). To support the molecular identification, fungal endophyte taxa were confirmed by inducing sporulation on PDA or V8 plates and using reported morphological criteria for identification of fruiting bodies structure and shape (*e.g.*, [82]–[83]). The specific accession numbers of our set of new fungal endophyte isolates obtained from *Gossypium hirsutum* are also shown in Table 2.

### Endophyte Community Analyses

To quantify fungal endophyte species diversity within samples or sites (α−diversity), we calculated the Shannon-Wiener biodiversity index (H′) using the frequency of isolation of fungal taxa per variety, tissue, time of the season and location using EstimateS software [37]–[38]. In order to statistically compare H′ values across samples we used bootstrapping with replacement (1000 iterations) to generate 95% confidence intervals for each H′ value [37]–[38]. In addition, to assess species richness and determine if our sampling intensity was sufficient, EstimateS was used to calculate fungal endophyte taxa accumulation curves using 1000 randomizations separately for leaves and squares/bolls. The curves were plotted with data from all fungal taxa represented by one or more isolates obtained from each plant tissue per location [37]–[38].

We compared variation in community composition and structure among varieties, times of the season, tissues and locations (β-diversity) using three different ecological community similarity measures. Multiple different pairwise similarity measures were examined because they consider different kinds of information that can lead to different insights when comparing communities [40]. We calculated (i) the Jaccard’s index comparing fungal taxa presence or absence among samples, (ii) the Bray-Curtis coefficient, which compares fungal taxa presence or absence as well as abundance among samples, and (iii) the Euclidean distance, which incorporates both quantitative abundance data and joint absences using total number of fungal taxa isolated. These parameters were calculated and cluster analyses performed using BOOTCLUS software to identify patterns and objectively determine groupings of multivariate data [39].

Matrices obtained by the cluster analyses of pairwise similarity measures of fungal endophyte communities were represented using non-metric multidimensional scale (NMDS) plots. Multidimensional scaling is designed to graphically represent relationships between objects in multidimensional space. The Kruskal’s stress value is used to decide which grouping of the data, depending on the number of dimensions used, is the most accurate (commonly acceptable when it is <0.2) [40]. NMDS is a robust visual analysis method applicable to a range of data types, it is amenable to several user-defined standardizations and transformations of the data, flexible in terms of which dissimilarity or similarity measure is used, and can be used for describing patterns and testing *a priori* hypotheses [40].

## Supporting Information

Figure S1 **Three dimensional NMDS plots of the effects of region, time of season, and tissue on whole fungal endophyte communities.** Three dimensional plots and associated Kruskal’s stress values of endophyte community comparisons as shown in the two dimensional plots in Figure 7. Results indicate no major change in observed clustering patterns with increased dimensionality despite increased confidence based on reduced Kruskal’s stress values (<0.2).
(TIFF)
