# Supplementary figures and images for: Spatial and Temporal Variation in Fungal Endophyte Communities Isolated from Cultivated Cotton (Gossypium hirsutum)
Source: PLoS One. 2013 Jun 11;8(6):e66049. doi: 10.1371/journal.pone.0066049 (PMC3679028; doi:10.1371/journal.pone.0066049)

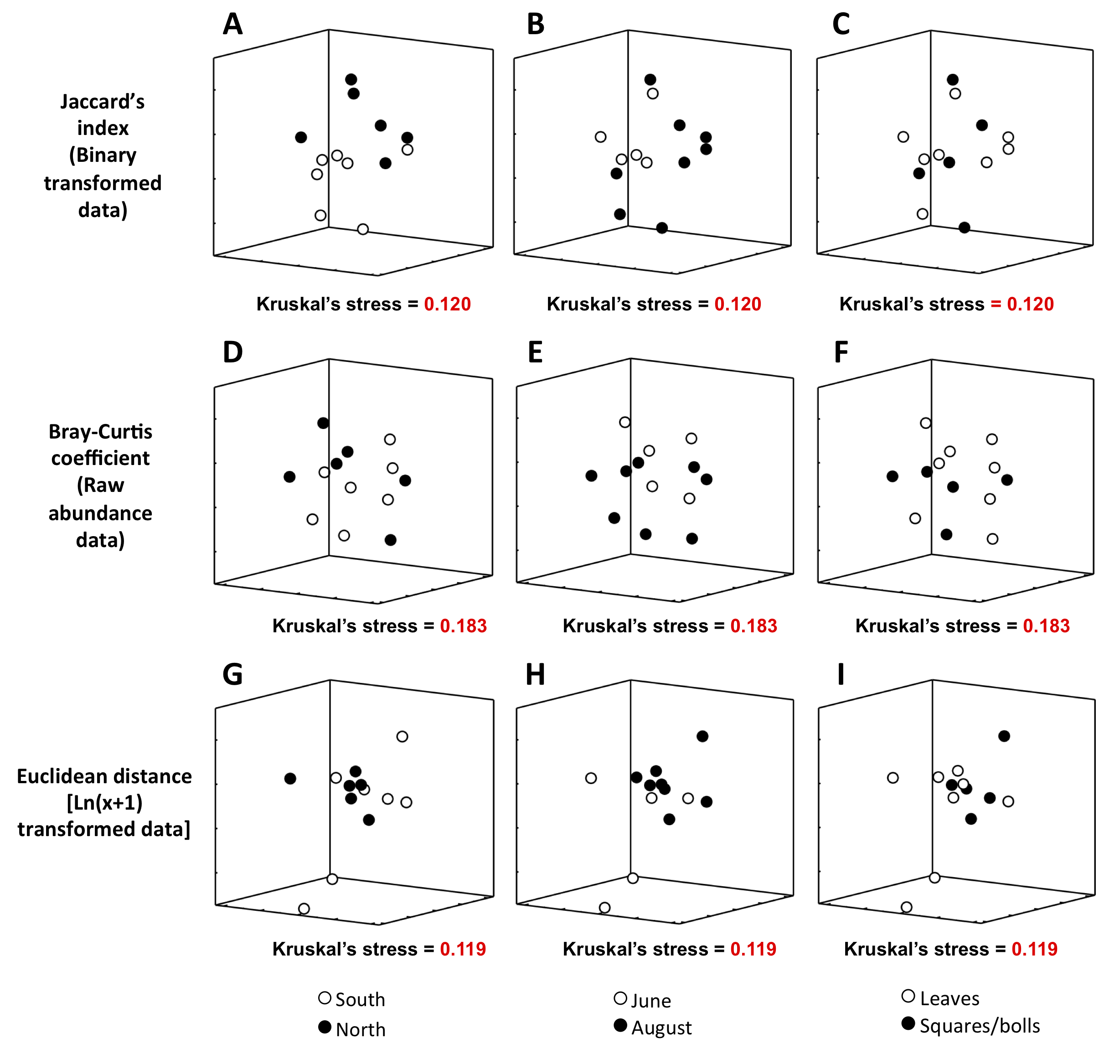

Supplement: Figure S1 — Three dimensional NMDS plots of the effects of region, time of season, and tissue on whole fungal endophyte communities. Three dimensional plots and associated Kruskal’s stress values of endophyte community comparisons as shown in the two dimensional plots in Figure 7. Results indicate no major change in observed clustering patterns with increased dimensionality despite increased confidence based on reduced Kruskal’s stress values (<0.2). (TIFF) [file pone.0066049.s001.tiff]
